# Vitamin D and Immunoglobulin E Status in Allergic Rhinitis Patients Compared to Healthy People

**DOI:** 10.25122/jml-2020-0015

**Published:** 2020

**Authors:** Haitham Alnori, Fawaz Abdulghani Alassaf, Mohanad Alfahad, Mohannad Emad Qazzaz, Mahmood Jasim, Mohammed Najim Abed

**Affiliations:** 1. Department of Surgery, College of Medicine, University of Mosul, Mosul, Iraq; 2. Department of Pharmacology, College of Pharmacy, University of Mosul, Mosul, Iraq; 3. Department of Pharmaceutics, College of Pharmacy, University of Mosul, Mosul, Iraq; 4. Department of Pharmacognosy and Medicinal Plants, College of Pharmacy, University of Mosul, Mosul, Iraq; 5. Department of Pharmaceutical Chemistry, College of Pharmacy, University of Mosul, Mosul, Iraq; 6. Department of Pharmaceutical Chemistry, College of Pharmacy, University of Mosul, Mosul, Iraq

**Keywords:** Allergic rhinitis, vitamin D and allergic rhinitis, IgE and allergic rhinitis, vitamin D and IgE, vitamin D, IgE and allergic rhinitis

## Abstract

Allergic rhinitis (AR) is a type of inflammatory condition that includes a group of symptoms, mainly affecting the nasal mucosa. Nasal obstruction, sneezing, stuffy or runny nose, in addition to swollen, itchy, red and watery eyes are the most common symptoms of the disease. These symptoms are triggered as a result of increased inflammatory mediators such as histamine and leukotrienes. Studies have recently shown the role of vitamin D (vit.D) in many allergic and immune conditions, where receptors for the active form of vit.D (1,25-dihydroxyvitamin D3) have been discovered on the surface of almost all types of inflammatory cells. Therefore, the present study was conducted to explore the level of vit. D in AR patients and its correlation with the severity of the disease. Two groups participated in the study; the first group included 49 patients who were diagnosed in a private otolaryngology clinic by the first author as having allergic rhinitis (AR group). The second one served as a control group and included 50 apparently healthy volunteers with no history of AR. The mean level of IgE and vit. D was found to be 326.3 and 10.2 ng/ml in the AR group, respectively, and 30.8 and 23.3 ng/ml in the control group, respectively. Ninety-three percent of AR patients have shown a deficiency in vit. D level, where 56% of this group showed severe deficiency. On the other hand, 34% of the control group has shown an insufficient level of vit. D. Additionally, 64% of AR patients have shown serum levels of IgE at values ranging between 100-299 ng/ml. Higher serum levels of IgE at values ranging between 300-599 ng/ml and 600-1000 ng/ml were observed in 25% and 11% of AR patients, respectively. The prevalence of low levels of vit. D in the AR group was significantly higher than that in the control group (P < 0.001). Vit. D deficiency is significantly related to severe AR symptoms and measuring serum vit. D level is recommended in the management plan of this group of patients.

## Introduction

Allergic rhinitis is an inflammatory condition of the nasal mucosa. Typical symptoms include nasal obstruction/ congestion, itching, watery nose, and sneezing [1]. Although it mainly affects the nose, AR is now considered a component in the diseases of the entire respiratory tract. AR affects about 10-20% of the global population, with around 500 million patients worldwide [2]. However, the prevalence of the disease differs between countries and is related to genetic, geographic, and climate factors and the types of allergens in a specific region [3]. In Iraq, a study was conducted by Alsamarai et al. [4] to test the correlation between AR and asthma in the Iraqi population, based on evidence that many asthmatic patients also suffer from AR [5]. The study showed that over 60% of asthmatic patients suffer from AR, and 6% of the non-asthmatic control population from Iraq have AR [4].

Traditionally, AR was classified as seasonal (symptoms appearing in a particular season) or perennial (symptoms throughout the year). This classification is no longer employed since some allergens may be seasonal in some regions and perennial in others, and many patients have multiple seasonal attacks throughout the year [6]. A more recent classification is based on symptoms’ duration (intermittent and persistent) and severity (mild and moderate to severe). Intermittent AR is defined as symptoms occurring for less than four weeks at a time, while in the persistent class, the patient is suffering for most of the year. Mild symptoms involve those that do not interfere with the patient’s ability to sleep and function normally. If sleep is significantly affected, and the patient becomes morbid, then the symptoms are moderate to severe [5]. 

Symptoms of AR are triggered by inflammatory mediators such as histamine and leukotrienes released as a result of increased immunoglobulin E (IgE) production from plasma cells. This increased production of IgE is mediated by cytokines released from inflammatory T cells invading the mucosa of the nasal cavity in response to the exposure of the mucosa to exogenous allergens [6]. Vit. D is a fat-soluble vitamin that is well known for its role in calcium homeostasis and bone integrity. More recently, studies have shown the role of vit.D in many allergic and immune conditions, where receptors for the active form of vit. D (1, 25-dihydroxyvitamin D3) have been discovered on the surface of almost all types of inflammatory cells and this has linked vit.D to immunity and immune diseases [7, 8].

Regarding the specific role of vit. D in AR, a literature search has shown a discrepancy in findings. For example, Wjst and Hyppönen found that the incidence of AR increases with serum levels of vit. D in Finland [9]. Similarly, it was found that the incidence of AR was higher in adults who have received vit. D supplementation during infancy [10]. Conversely, Erkkola et al. observed that maternal vit. D intake reduces the risk of AR in children at the age of 5 years [11]. Another study conducted in Iran showed that the prevalence of severe vit. D deficiency was much higher in patients with AR [12]. Likewise, it was found by Sudiro et al. that vit. D deficiency can be related to AR and its severity in Indonesia [3].

Accordingly, this study aims at examining the correlation between vit. D levels and AR in a sample population of the city of Mosul, Nineveh province in the north of Iraq.

## Material and Methods

### Study design and methodology

This study was conducted in Mosul city from March to October 2018 and involved two groups; the first group comprised AR patients diagnosed in a private fully equipped otolaryngology clinic by the first author as having allergic rhinitis (AR group). Forty-nine patients were clinically diagnosed, and nasal endoscopy was performed on every patient to exclude other conditions such as sinusitis, nasal polyposis, and nasal septal deviation. The diagnosis was made according to the “AR and its Impact on Asthma” (ARIA) guidelines, a runny nose and nasal obstruction as the main complaints [5]. The second group included 50 apparently healthy volunteers with no history of AR, aged between 20 and 50 years, and regarded as the control group. IgE was measured in both groups. Both groups enrolled had blood tests in order to determine their 25-hydroxyvitamin D3 serum levels. The study was approved by the Health Research Ethics Committee at the College of Medicine, University of Mosul (No: UOM/COM/2019/2). A written consent form was provided for each subject participating in the study with full awareness of the details. Blood samples were collected from all the subjects, and 25-hydroxyvitamin D3 serum level was measured by the immunoassay method using the Dimension® Suite from Siemens. A vit. D level of less than 10 ng/ml was considered severe vit. D deficiency, whereas a vit. D level of 10-12 ng/ml was considered moderate vit.D deficiency, while a vit. D level of 12.1-20 ng/ml was regarded as vit. D insufficiency; finally, a serum level of more than 20 ng/ml was considered normal [13]. None of the participants in both groups was receiving vit. D supplements. The Serum IgE level was measured by the immunoassay method (Allegro, Algeria). AR is classified as intermittent if symptoms are present in less than four days a week or less than four weeks a time. Perennial AR means symptoms persist for four days or more a week and four weeks or more a time. AR is classified as moderate to severe if one or more of the following is present: abnormal sleep, impairment of daily activity, abnormal work at school, or troublesome symptoms. If none of the above symptoms is present, it is regarded as mild AR [5].

Inclusion criteria included patients with AR attending the private clinic of the first author with age ranging between 18-55 years.

Exclusion criteria involved AR patients who have a body mass index (BMI) greater than 26 kg/m2, patients with inflammatory or immunological conditions such as asthma, nasal polyposis, and rheumatoid arthritis, patients with chronic illnesses such as diabetes mellitus, renal insufficiency, and abnormal vit. D metabolism, in addition to patients receiving chronic or recent therapy with steroids, antihistamines, vit. D supplements and chemotherapeutic agents.

### Data analysis

All values were expressed as the mean ± standard deviation (SD) or standard error of mean (SEM) where indicated. Student’s paired t-test for single data comparison was performed. Pearson’s correlation was used to analyze the relationship between the studied parameters. GraphPad Prism 8.0 software was utilized to assess the statistical significance (P < 0.05) of any difference between the mean values.

## Results

The studied sample was divided into two groups: the AR group included AR patients of the same age, sex and weight as the control group, which included healthy volunteers. Characteristics of the studied population are summarized in Table 1, which represents the age, sex, weight, and BMI status of the studied population.

**Table 1:** Characteristics of the studied groups.

Studied sample	Age (years)	Number (M/F)	Weight (kg)	BMI (kg/m^2^)
Control group	30.57 ± 6.5	50 (22/28)	71.1 ± 7.69	24.7 ± 1.44
AR group	28.1 ± 9.9	49 (20/29)	70.7 ± 18	25.8 ± 6

Results are expressed as mean ± S.D.; M: male, F: female.

Most patients in the AR group (71%) had moderate to severe persistent AR. At the same time, the mild intermittent class was shown to be the least presented when compared to the other investigated classes of the disease, as shown in Table 2.

**Table 2:** Classification of AR group according to severity.

Class	% (N) of patients affected
**Mild intermittent**	2.04 (1)
**Mild persistent**	6.12 (3)
**Moderate-severe intermittent**	20.4 (10)
**Moderate-severe persistent**	71.42 (35)

(N)= Number of patients.

**Figure 1:**
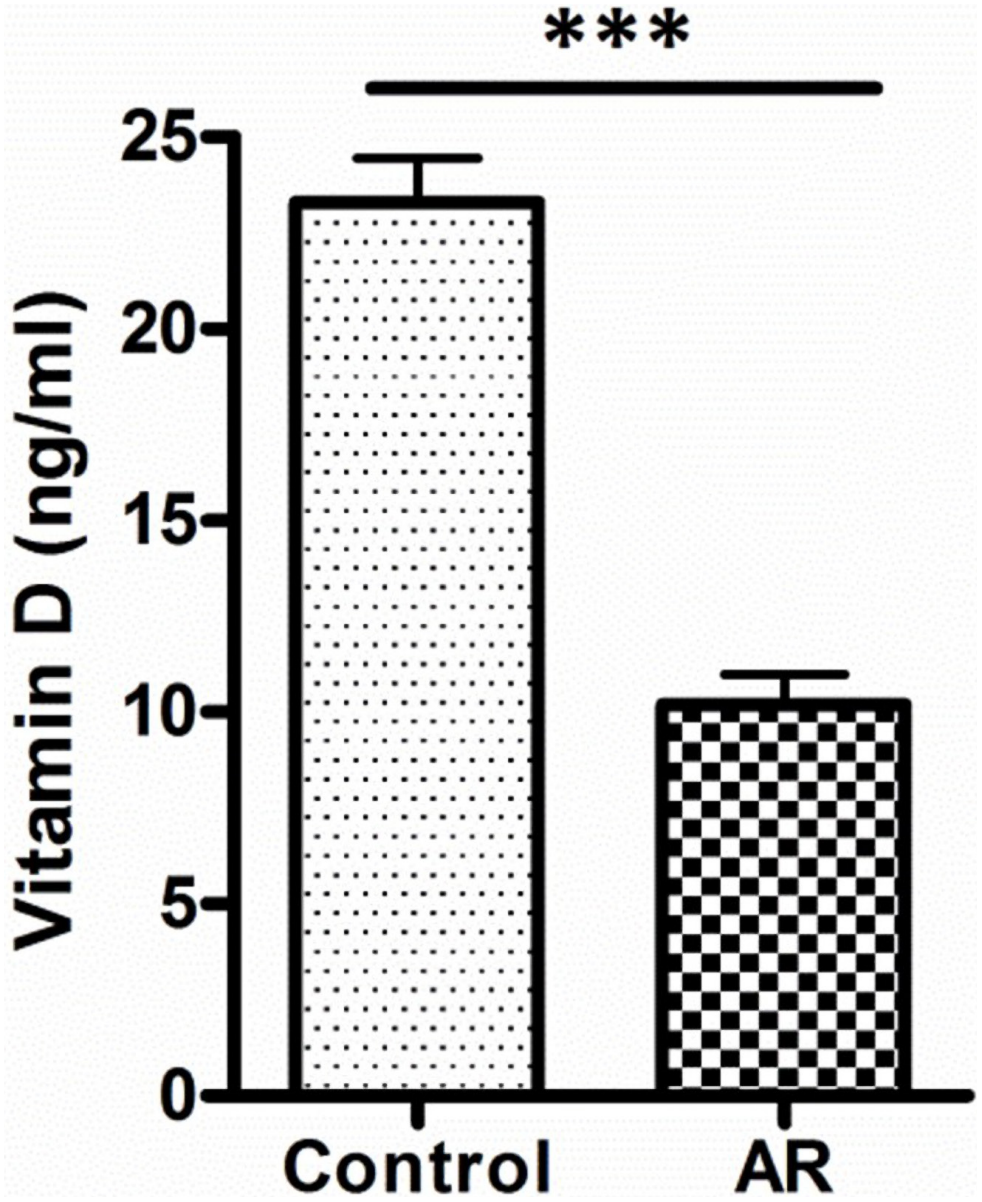
Serum vitamin D level in the studied groups. Data are expressed as mean ± SEM. ***P < 0.001 represents a difference of statistical significance between AR and control group. Student’s paired t-test was used for statistical comparison.

**Figure 2:**
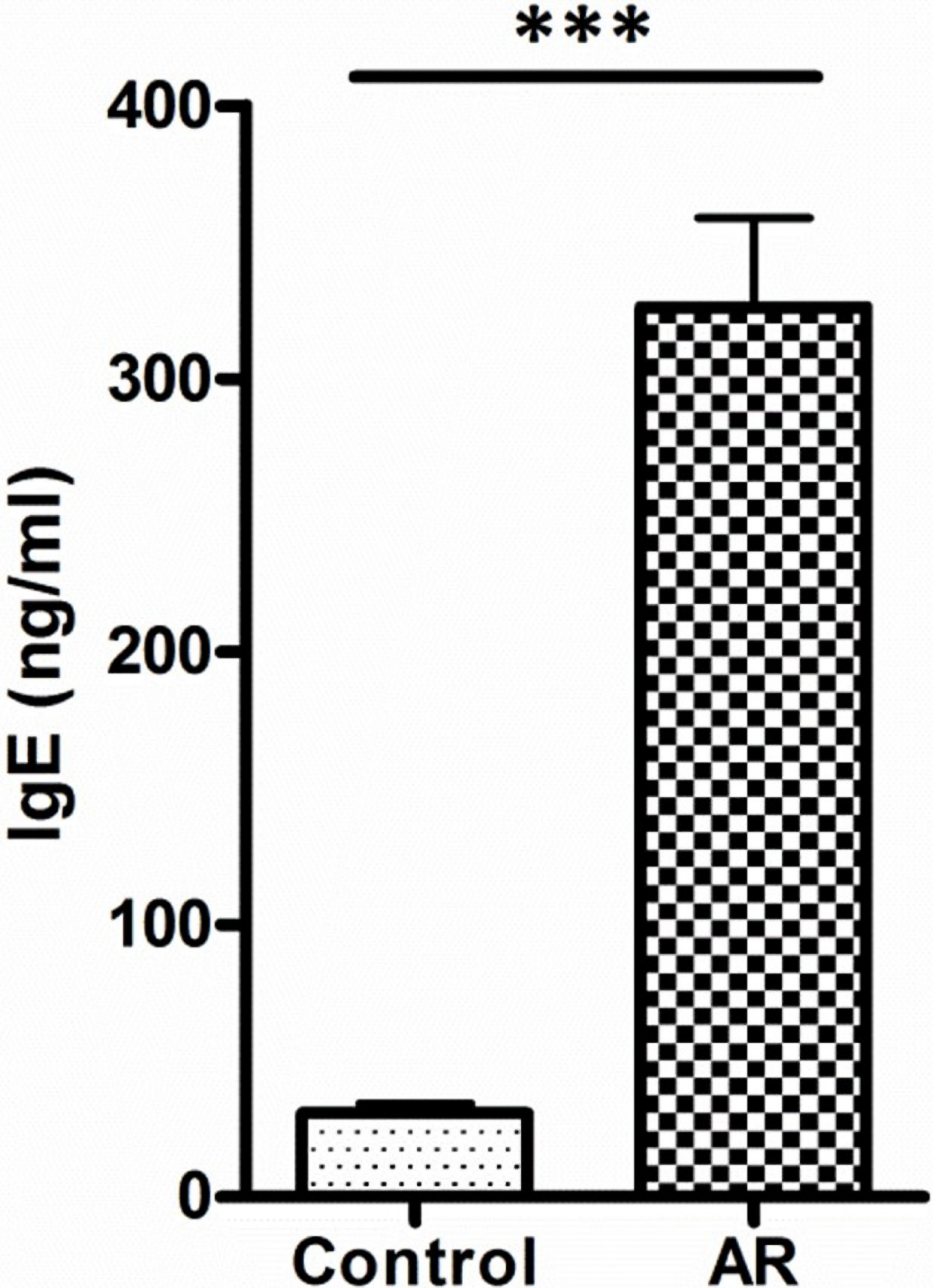
Serum IgE level in the studied groups. Data are expressed as mean ± SEM. ***P < 0.001 represents a difference of statistical significance between AR and control group. Student’s paired t-test was used for statistical comparison.

**Figure 3:**
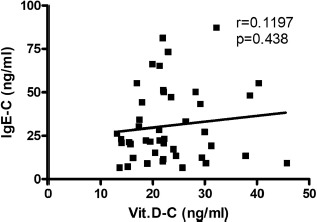
Serum IgE level versus serum vitamin D level in the control group. (r): Pearson correlation coefficient between the two investigated variables. Statistically non-significant correlation is shown at P > 0.05. IgE-C and Vit.D-C: Serum level of IgE and vit. D in the control group.

**Figure 4:**
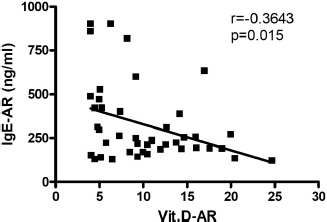
Serum IgE level versus serum vitamin D level in AR group. (r): Pearson correlation coefficient between the two investigated variables. Statistically significant negative correlation is shown at *P < 0.05. IgE-AR and Vit.D-AR: Serum level of IgE and vit. D in the AR group.

**Figure 5:**
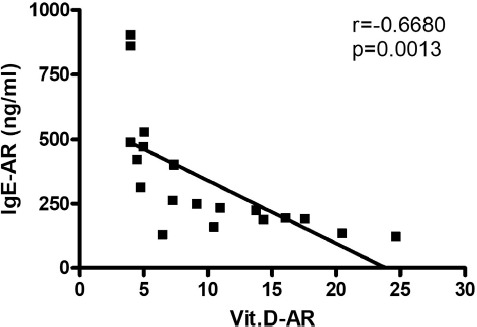
Serum IgE level versus serum vitamin D level in patients with moderate-severe persistent AR. (r): Pearson correlation coefficient between the two investigated variables. Statistically significant negative correlation is shown at **P < 0.01. IgE-AR and Vit.D-AR: Serum level of IgE and vit. D in the AR group.

The mean level of IgE and vit. D was found to be 30.8 and 23.3 ng/ml, respectively, in the control group and 326.3 and 10.2 ng/ml, respectively, in the AR group. Ninety-three percent of AR patients have shown a deficiency in vit. D levels, where 56% of this group showed severe deficiency. On the other hand, 34% of the control group has shown an insufficient level of vit. D. Sixty-four percent of AR patients have shown serum level of IgE at values ranging between 100-299 ng/ml, while 25% and 11% of AR patients have shown higher serum levels of IgE at values ranging between 300-599 ng/ml and 600-1000 ng/ml, respectively. The prevalence of low levels of vit. D in the AR group was significantly higher compared to the control group (P < 0.001), as shown in Figure 1. The serum level of IgE in the AR group was significantly higher than that in the control group (P < 0.001), as illustrated in Figure 2. The correlation of serum levels of IgE with vit. D in the control group was found to be statistically insignificant (P > 0.05). The correlation coefficient (r) between the variables was 0.1197, which does not reflect a significant correlation between the two variables (Figure 3). However, a statistically significant negative correlation was observed between the serum IgE and vit. D levels in the AR group (P < 0.05). The correlation coefficient (r) between the variables is -0.3643, which reflects the negative correlation between the two variables (Figure 4). To further link the severity of AR to vit. D deficiency, serum IgE level in the moderate-severe class (which represents the majority of AR patients in this study) was correlated to the vit. D level and the results have shown a stronger negative correlation between the studied variables at r=-0.6680 and a P-value of less than 0.01 (Figure 5).

## Discussion

Vitamin D is an essential nutrient required for healthy bones and the immune system. It has two major forms: (D2; ergocalciferol and D3; cholecalciferol); both forms can be obtained from foods. However, only vitamin D3 can be made by the human body [14]. The parameter that is directly tested to measure vitamin D3 level in the blood is 25(OH)D3. However, 1,25(OH)2D3 is the most biologically active metabolite of vitamin D3 [15].

The association between immune diseases and 1,25(OH)2D3 had been documented since 1984. A link between allergic disorders (especially asthma) and vit. D levels had been reported in many epidemiological studies [16]. Several mechanisms were reported to be involved in the immune modulation effect of 1,25(OH)2D3 on immune cells and some cytokines. Among these mechanisms, T-cell proliferation inhibition, suppressing the differentiation and transcription of Th17 cells, enhancing Th2 cell development, decreasing macrophage inflammation, T-cell stimulation and inhibiting immunoglobulin secretion, including IgE secretion can be noted [17]. The immunoregulatory effect of vitamin D3 provides a good base for a correlation between AR and vitamin D3 serum level; AR pathogenesis comprises phenotype transfer of Th1 to Th2 in the production of CD4+ T cells in addition to the involvement of Th17 and T-reg cells in the disease course. Induction of Th1 shift to Th2 by augmenting Th2 development and inhibition of T cell proliferation are the main immunomodulatory actions of vitamin D3 on top of subdues processes of differentiation and transcription of Th17 cells and aids the stimulation of Foxp3+ T-reg cells [18].

Age, weight, type of food, skin pigment, lifestyle, residence and sun exposure are factors that could affect vit. D levels in one way or another. Elderly, as well as overweight or obese individuals, usually have low vit. D levels. Also, diets low in fish and dairy are associated with vit. D deficiency. People with dark skin, persons frequently using sunscreen, wearing long sleeve shirts, head cover, traveling by car, having a sedentary lifestyle, limited availability of the sun in the living area, as well as variation in sun exposure due to season variation, time of the day exposure and atmospheric components, could all affect the vit. D status [19, 20]. Iraq is a subtropical country, where summer is sunny, and winter is mostly cloudy. Vit. D deficiency is expected to be prevalent during winter, so we performed our study during spring, summer and autumn in order to avoid this effect.

This study was directed to explore the relationship between vit. D serum level and the prevalence of AR in both patients and healthy subjects. Total serum IgE is not helpful for the diagnosis of AR according to the ARIA guidelines. Despite this, IgE was investigated in this study. Our hypothesis to include IgE was that vit. D is an immunomodulator, and its deficiency can lead to an increased allergic response. Therefore, IgE may be associated with vit. D deficiency for the same reason.

The study revealed that 93% of patients diagnosed with AR had a deficiency in vit. D levels. We also found an association between IgE levels and vit. D deficiency in these patients; this association was found to be stronger in patients with moderate to severe persistent AR.

The present study showed a significant difference in the mean serum level of vit. D between the healthy group and the AR group (p < 0.001). The statistics revealed that most of the patients were diagnosed as having the severe class of the disease (91.8%); the low number of patients in our study with mild AR (intermittent - 1 patient, mild to severe - 3 patients) may be explained by the fact that these patients commonly depend on self-prescribed medications due to mild symptoms.

Our study showed that the prevalence of vit. D deficiency is obvious among the AR group, which matches the findings of Sudiro et al., who reported a correlation between the severity of vit. D deficiency and the severity of AR. In addition, Vatankhah V. et al. described the AR group as a vit. D deficient group in comparison to people with normal vit. D levels [3, 21]. However, another study reported no correlation between vit. D levels and the severity of AR [22].

Hypovitaminosis D was reported in many literature studies conducted in the Middle East, revealing that, despite the plentiful sunny climate in this part of the world, the region registers low vit. D levels among different age groups. Factors like insufficient vit. D intake during infancy, economic causes, inadequate sun exposure, traditional clothing style, and urban living may contribute to the low level of vit. D. The findings of our study are consistent with these global annotations [23].

The serum concentrations of vit. D that are associated with deficiency, adequacy, and optimum overall health are still tremendously questionable. The Institute of Medicine (IOM) states that the serum concentrations of vit. D less than 12 ng/ml may predispose people to a risk of vit. D deficiency. Serum levels between 12–20 ng/ml predispose to a potential risk of inadequacy. Almost everyone is considered to have appropriate vit. D levels at serum values greater than 20 ng/ml. Additionally, the committee of IOM reported that 20 ng/ml is the amount of serum vit. D serving the needs of about 97.5% of the population. Additionally, serum levels greater than 50 ng/ml may associate with possible adverse effects [13].

Our study suggests that vit. D level is one of the parameters that have to be checked in AR patients, in accordance with the findings of other similar studies which correlate vit. D deficiency to AR [3, 24]. The results of the present study also support the recommendations to adjust vit. D status among different age groups [23]. Detailed information about the lifestyle of the participants and analysis of the data concerning factors affecting vit. D serum levels, either directly or indirectly, are considered a limitation of this study.

## Conclusion

Vit. D deficiency is linked with the severity of AR, and monitoring serum vit. D levels is advisable in this group of patients. In addition, adjustment of vit. D levels is sensible in apparently healthy people as the results of this study have found a subclinical level of vit. D in a significant number of healthy volunteers. Moreover, future studies are recommended to investigate the role of the administration of vit. D as an add-on therapy for AR patients that have a low level of vit. D.

## Conflict of Interest

The authors declare that there is no conflict of interest.
